# Radiotherapy Planning System to Measure Tumor Doubling Time in Cervical Cancer

**DOI:** 10.7759/cureus.12612

**Published:** 2021-01-10

**Authors:** Hiromasa Kurosaki, Nobuko Utsumi, Kosei Miura

**Affiliations:** 1 Department of Radiation Therapy, Japan Community Healthcare Organization, Tokyo Shinjuku Medical Center, Tokyo, JPN; 2 Department of Radiation Oncology and Proton Medical Research Center, University of Tsukuba, Tsukuba, JPN

**Keywords:** tumor doubling time, cervical cancer, radiotherapy planning system

## Abstract

Tumor doubling time is an important clinical parameter, but it is rarely reported in cervical cancer. We encountered a case in which the tumor doubling time could be measured using a radiotherapy planning device. A woman in her 40s was diagnosed with cervical cancer stage IB1 (squamous cell carcinoma) and refused treatment. One year and five months later, definitive radiation therapy was administered. Magnetic resonance imaging was performed five times before the start of treatment. When the tumor volume was measured using the radiotherapy planning system - RayStation🄬 (RaySearch Laboratories, Stockholm, Sweden) on the T2 sagittal image, the tumor doubling time was 76 days, and the tumor volume had increased exponentially.

## Introduction

Pathological characteristics of malignant tumors include the invasive growth and metastasis formation. In addition, the growth rate related to the appearance over time is also an important indicator of the characteristics of malignant tumors. Cancer cell cycle time, cancer cell doubling time (DT), and tumor volume (TV) DT are examples of tumor growth rates. The DT is a prognostic factor in various tumors [1,2].

From the 1950s to 1970s, computed tomography (CT) and magnetic resonance imaging (MRI) were rarely used in the determination of the DT. Therefore, estimated TV from chest radiographs was used in clinical practice [3-5]. In recent reports, CT or echo has often been used [6-8]. On the other hand, in radiotherapy, the radiotherapy planning system (RPS) has been developed so that the TV can be measured from various image modalities. Herein, we report a case of cervical cancer in which the DT was measured from MRI images using RayStation🄬 (RaySearch Laboratories, Stockholm, Sweden), a type of RPS.

## Case presentation

**Figure 1:**
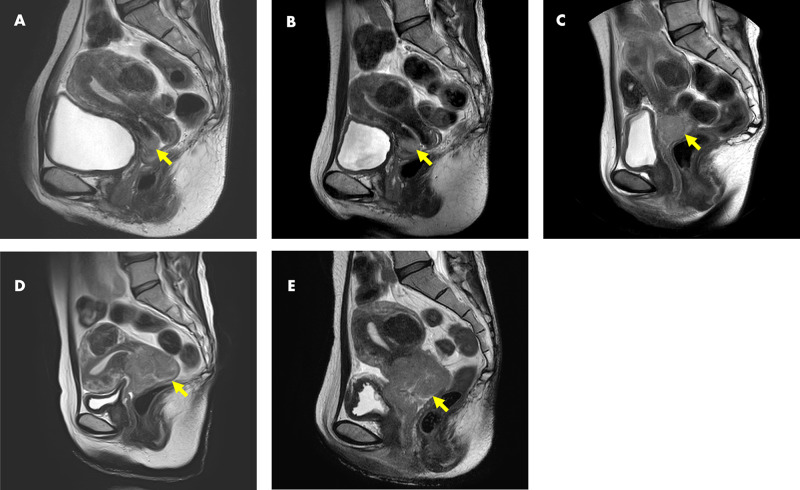
Image of magnetic resonance imaging T2 sagittal: (A) first time (Day 0), (B) Day 126, (C) Day 216, (D) Day 427, and (E) Day 512.

A woman in her 40s was diagnosed with cervical cancer stage IB1 (squamous cell carcinoma), but refused treatment. One year and five months later, definitive radiotherapy was performed at cancer stage IIIB. Five MRI scans (days 0, 126, 216, 427, and 512) were taken before treatment was started (Figure 1).

The TV was measured using RayStation🄬. Two radiation oncologists collaborated to contour the tumor using MRI T2 sagittal images. The following formula for calculating the DT proposed by Schwartz was used [9]:

 DT=(t_2_-t_1_)*ln(2)/ln(q_2_/q_1_) (TV at time t_1_ is q_1_, and TV at time t_2_ is q_2_)

**Figure 2:**
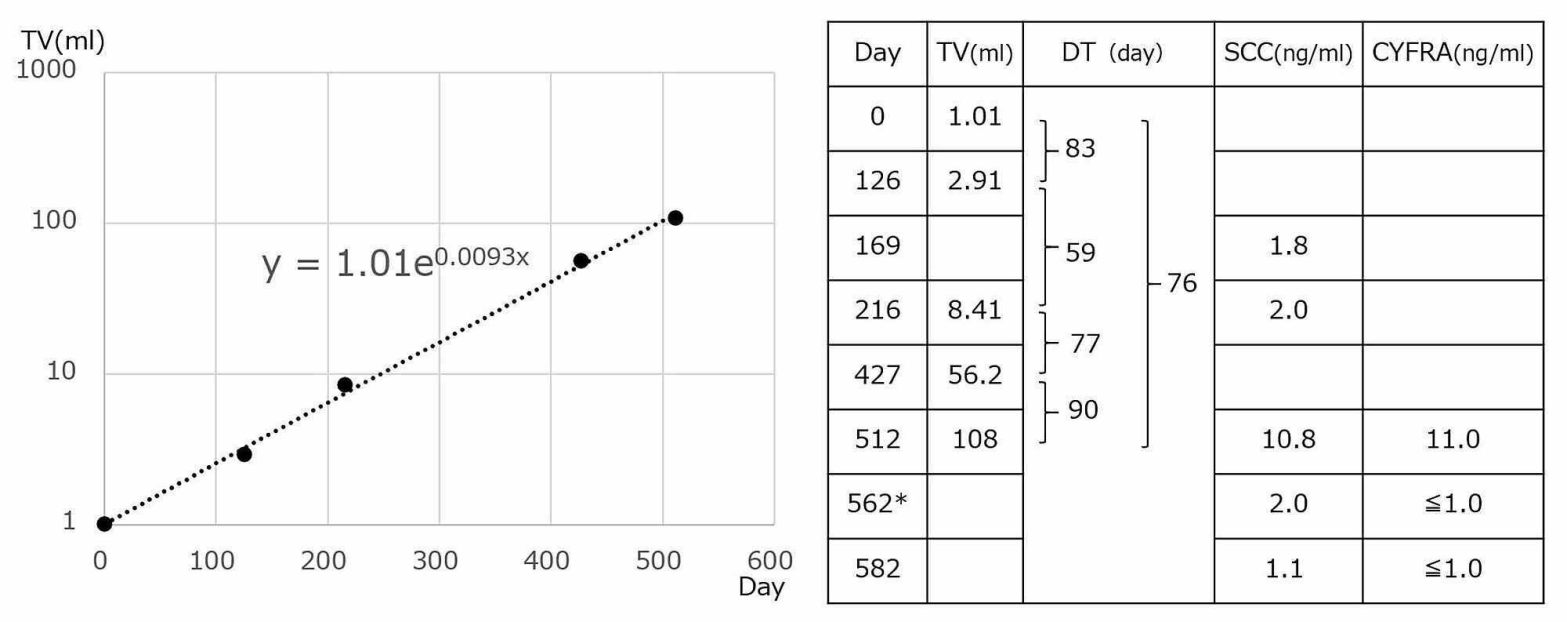
Course of the tumor volume. The DT for the entire period was 76 days. DT: tumor doubling time, SCC: squamous cell carcinoma antigen in the serum, CYFRA: cytokeratin 19 fragment in the serum *: Radiation therapy end date

The TV increased almost exponentially (Figure 2), with a total tumor DT of 76 days.

Tumor markers were normalized on the last day of radiation therapy (Day 562), and no mass was found on pelvic examination.

## Discussion

In 1956, Collins et al. measured lung metastases over time and reported that their growth was exponential [3]. They measured the tumor diameter with a chest radiograph, and by calculating using the formula:

 DT=(t_2_-t_1_)*ln(2)/3*ln(d_2_/d_1_) (tumor diameter at time t_1_ is d_1_, and tumor diameter at time t_2_ is d_2_）　

reported that the tumor growth curve became exponential. Reports of that era measured the diameter of the tumor and regarded the tumor as a sphere to calculate the DT. Thereafter, the DT was reported by calculating the TV using the major axis and minor axis of a CT image instead of chest radiographs [1]. On the other hand, the TV determined using the 2D and 3D methods show a strong correlation, although the 3D method is known to be more accurate [10].

Reports of the DT in cervical cancer are limited. Sugawara measured 10 lung metastases from cervical cancer in eight patients and reported a DT of 49 days (21-144 days) [5]. In addition, Sugawara reported that it was better to increase a single dose for tumors with a DT of 40 days or more radiobiologically. In cervical cancer, high-dose irradiation by intracavitary irradiation has been clinically performed for a long time, and it can be said that its biological theory has been explained again.

In cervical cancer, instead, studies using bromodeoxyuridine labelling index were reported [11,12]. On the one hand, a report by Tang et al. concluded that there is no advantage in clinical outcome when either bromodeoxyuridine labelling index or potential DT is used in both multivariate analysis and univariate analysis [11]. They explained the reason is that flow cytometry analysis is easily affected by the contamination of non-tumor cells, and its result is needed to correct the division of the cells passing through mitotic division especially for the case of diploid tumor.

In this case, the patient refused treatment. In this pattern, patients miss their opportunities to have proper treatments due to the delay of diagnosis. We should explain to those patients who refuse treatment that their tumors will grow a hundredfold in a year and a half.

The CT cannot distinguish the difference between a tumor and normal uterus, and the macroscopic TV is not clear without MRI; consequently, the DT for cervical cancer has not been reported. Moreover, even if the tumor is detected by MRI, the shape of the cervical cancer is not necessarily spherical, and it is impossible to calculate the TV with the tumor diameter alone. In addition, only axial images can be accepted on some conventional treatment planning system (TPS) due to the limitation of the systems. In contrast, sagittal images, which clearly tell us cervical cancer, can be used for contouring on RayStation🄬. RayStation🄬 was used to calculate the dose of radiotherapy to contour the tumor for the calculation of various TVs and to combine various images with the concept of non-rigid image registration [13]. Although there are reports of using CT workstations [8], RPS can also measure the DT from two MRI images.

## Conclusions

We encountered a case of cervical cancer whose DT could be measured from MRI T2 sagittal images using RPS. Tumor growth was exponential, and the DT for cervical cancer, in this case, was 76 days. This TV increased a hundredfold in a year and a half.
